# Genetic Analysis of the Individual Contribution to Virulence of the Type III Effector Inventory of *Pseudomonas syringae* pv. *phaseolicola*

**DOI:** 10.1371/journal.pone.0035871

**Published:** 2012-04-27

**Authors:** Alberto P. Macho, Adela Zumaquero, Juan J. Gonzalez-Plaza, Inmaculada Ortiz-Martín, José S. Rufián, Carmen R. Beuzón

**Affiliations:** Department of Biología Celular, Genética y Fisiología, Instituto de Hortofruticultura Subtropical y Mediterránea, Universidad de Málaga-Consejo Superior de Investigaciones Científicas (IHSM-UMA-CSIC), Málaga, Spain; Max Planck Institute for Chemical Ecology, Germany

## Abstract

Several reports have recently contributed to determine the effector inventory of the sequenced strain *Pseudomonas syringae* pv. *phaseolicola* (*Pph*) 1448a. However, the contribution to virulence of most of these effectors remains to be established. Genetic analysis of the contribution to virulence of individual *P. syringae* effectors has been traditionally hindered by the lack of phenotypes of the corresponding knockout mutants, largely attributed to a high degree of functional redundancy within their effector inventories. In support of this notion, effectors from *Pseudomonas syringae* pv. *tomato* (*Pto*) DC3000 have been classified into redundant effector groups (REGs), analysing virulence of polymutants in the model plant *Nicotiana benthamiana.* However, using competitive index (CI) as a virulence assay, we were able to establish the individual contribution of AvrPto1*_Pto_*_DC3000_ to *Pto* DC3000 virulence in tomato, its natural host, even though typically, contribution to virulence of AvrPto1 is only shown in strains also lacking AvrPtoB (also called HopAB2), a member of its REG. This report raised the possibility that even effectors targeting the same defence signalling pathway may have an individual contribution to virulence, and pointed out to CI assays as the means to establish such a contribution for individual effectors. In this work, we have analysed the individual contribution to virulence of the majority of previously uncharacterised *Pph* 1448a effectors, by monitoring the development of disease symptoms and determining the CI of single knockout mutants at different stages of growth within bean, its natural host. Despite their potential functional redundancy, we have found individual contributions to virulence for six out of the fifteen effectors analysed. In addition, we have analysed the functional relationships between effectors displaying individual contribution to virulence, highlighting the diversity that these relationships may present, and the interest of analysing their functions within the context of the infection.

## Introduction

*Pseudomonas syringae* is a gram-negative host-specific pathogen that depends on the Hrp type III secretion system (T3SS) to secrete and translocate effector proteins into the plant cell, thus causing disease in compatible hosts, and triggering a hypersensitive response (HR) in incompatible hosts [1]. The specificity of the T3SS-mediated plant-pathogen interaction is based on the set of effectors translocated by each strain [2]. Although the T3SS is essential for *P. syringae* infection, mutation of individual effectors has rarely succeeded in revealing virulence attenuation [3]–[7]. Frequently, reports showing effector contribution to virulence have resorted to the use of multiple effector mutants [8]–[10]. Thus, functional redundancy between effectors has been repeatedly proposed as the determining factor hindering the detection of virulence phenotypes for single effector mutants. In support of this notion, functional characterisation has already revealed seemingly similar defence suppressing capabilities for many *P. syringae* effectors [11]. In some cases, such as that of effectors AvrPto1 and AvrPtoB (now also called HopAB2) from *P. syringae* pv. *tomato* DC3000 (hereafter referred to as *Pto* DC3000), studies have demonstrated that the same defence signalling pathway is targeted by both effectors [12], [13]. In keeping with this, recent studies have classified the effectors from this strain into redundant effector groups (REGs), and established a minimal effector repertoire of eight as enough to render a functionally effectorless *Pto* DC3000 derivative, capable of full growth within the model plant *N. benthamiana* [10], [14]. On the other hand, virulence assays with higher sensitivity than standard assays, *i.e.* competitive index assays (CIs), have allowed detection of virulence phenotypes for single effector mutants such as AvrPto1 [15], [16].

In this work, we have carried out an extensive analysis of the effector inventory of the *P. syringae* pv. *phaseolicola* 1448a strain (hereafter *Pph* 1448a), using a collection of single effectors mutants previously generated by our team [17]. We have analysed symptom development for each strain, as well as growth in mixed infection (CI) at different stages after infiltration into *Phaseolus vulgaris* (common bean), its natural host. When CIs were used, we detected reproducible virulence phenotypes for six out of the fifteen single effector mutant strains analysed. Only one of these mutants was also accompanied by a delay in induction of disease symptoms. The virulence attenuation of the mutants can be complemented by expression of the corresponding effector from a plasmid. However, we also show that overexpression of some of these effectors can be detrimental for wild type growth. We also carried out CI assays following dip-inoculation into bean leaves for several of the mutant strains, obtaining very similar results to those generated using infiltration. Furthermore, we have analysed the functional relationships between effectors displaying individual contribution to virulence, applying a conceptual modification of CI analysis, the cancelled-out index or COI, to carry out genetic analysis of single and double mutants. COI analysis were previously developed for such purpose in animal pathogens [18], and have already been applied in *P. syringae* to establish functional relationships between regulatory elements of the type III secretion system [19], [20], and between plant defence signalling cascades triggered by heterologous effectors [21], [22].

## Results

### Development of symptoms by individual effector knockout mutants

*P. syringae* effectors are usually named as defined by the unified effector nomenclature [23], specifying the strain of origin as a sub-index after the effector name (e.g. HopR1_Pph1448a_). However, to avoid unnecessary repetitions, the name of the strain will only be specified when effectors from different strains are mentioned in the same context, using just the name of the effector when the strain of origin has been already stated.

**Figure 1:**
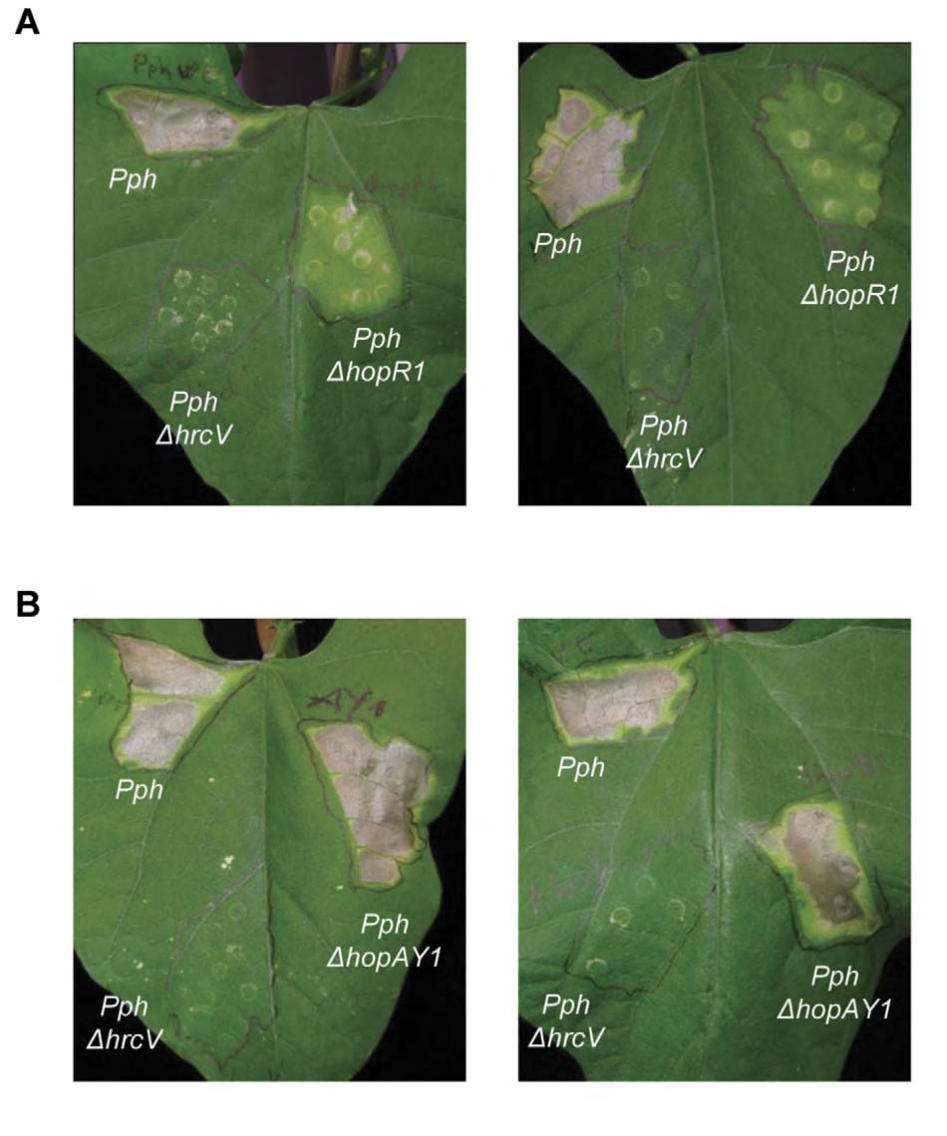
Disease symptoms in bean plants. Plants were infiltrated with 10^6^ cfu/ml, and symptoms documented at 7 days post-inoculation (dpi). **A**. Virulence symptoms caused by *Pph ΔhopR1* are significantly reduced in relation to wild type (*Pph*)-induced symptoms. **B**. *Pph ΔhopAY1* causes similar symptoms to those induced by wild type (*Pph*). The experiment was carried out thrice with similar results. Images show representative results.

We used a collection of fifteen mutants of *P. syringae* pv. *phaseolicola* (*Pph*) 1448a generated in our laboratory (Table 1) [17] to monitor the development of symptoms throughout time, following leaf infiltration. The mutant collection included thirteen effector mutants, and a mutant in *avrD1*, an HrpL-dependent gene encoding the enzyme syringolide [24], for which translocation through the T3SS is unclear [25], [26]). It also includes two genes currently considered to encode helper proteins that may or may not be translocated [27]–[29]. Only one effector knockout, Δ*hopR1*, displayed a sustained and reproducible reduction in the induction of disease symptoms in bean leaves whereas the rest either displayed no differences with the wild type strain, or differences too subtle or variable to be established with confidence (Table 2 and Figure 1). The absence of a virulence phenotype for the majority of the single mutants in these assays is in keeping with results obtained in previous analysis of single effector mutants in *P. syringae* strains [3]–[7].

**Table 1:** Bacterial strains used in this work.

Bacterial strain	Description	Antibiotic resistance	Source
*P. syringae* pv. *phaseolicola* 1448a	Race 6, wild-type strain	Nal^r^	[60]
*P. syringae* pv. *phaseolicola* 1449b	Race 7, wild-type strain		[60]
RW60	1449b derivative, Vir^−^, pAV511^−^	Rif^r^	[41]
IOM1	Δ*hrcV*	Nal^r^ Km^r^	[31]
IOM7	Δ*hrpL*	Nal^r^ Km^r^	[31]
AZJ19	Δ*hopAB1::nptII*	Nal^r^ Km^r^	[17]
AZJ21	Δ*avrB2::nptII*	Nal^r^ Km^r^	[17]
AZJ22	Δ*hopR1::nptII*	Nal^r^ Km^r^	[17]
AZJ23	Δ*hopAY1::nptII*	Nal^r^ Km^r^	[17]
AZJ24	Δ*hopAW1::nptII*	Nal^r^ Km^r^	[17]
AZJ25	Δ*hopAS1::nptII*	Nal^r^ Km^r^	[17]
AZJ26	Δ*hopAJ1::nptII*	Nal^r^ Km^r^	[17]
AZJ27	Δ*hopAU1::nptII*	Nal^r^ Km^r^	[17]
AZJ28	Δ*hopAK1::nptII*	Nal^r^ Km^r^	[17]
AZJ29	Δ*hopAE1::nptII*	Nal^r^ Km^r^	[17]
AZJ30	Δ*hopD1::nptII*	Nal^r^ Km^r^	[17]
AZJ31	Δ*hopQ1::nptII*	Nal^r^ Km^r^	[17]
AZJ32	Δ*hopG1::nptII*	Nal^r^ Km^r^	[17]
AZJ33	Δ*hopI1::nptII*	Nal^r^ Km^r^	[17]
AZJ34	Δ*avrD1::nptII*	Nal^r^ Km^r^	[17]
JRP4	Δ*hopAB1*	Nal^r^	This work
AZJ38	Δ*hopAB1* Δ*hopI1::nptII*	Nal^r^ Km^r^	This work
AZJ39	Δ*hopAB1* Δ*hopQ1::nptII*	Nal^r^ Km^r^	This work
AZJ41	Δ*hopAB1* Δ*hopR1::nptII*	Nal^r^ Km^r^	This work

**Table 2:** Analysis of symptom development after inoculation with different strains.

Strain	Genotype	Symptoms	Symptoms
		7–8 dpi	10–12 dpi
1448a	Wild type	****	******
IOM7	Δ*hrcV*		
AZJ19	Δ*hopAB1*	****	******
AZJ21	Δ*avrB2*	****	ND
AZJ22	Δ*hopR1*	*	***
AZJ23	Δ*hopAY1*	****(*)	******
AZJ24	Δ*hopAW1*	****	ND
AZJ25	Δ*hopAS1*	****	ND
AZJ26	Δ*hopAJ1*	****	******
AZJ27	Δ*hopAU1*	***(*)	******
AZJ28	Δ*hopAK1*	****	******
AZJ29	Δ*hopAE1*	****	******
AZJ30	Δ*hopD1*	****	ND
AZJ31	Δ*hopQ1*	****(*)	******
AZJ32	Δ*hopG1*	****	******
AZJ33	Δ*hopI1*	****	ND
AZJ34	Δ*avrD1a*	****	ND

(*) Affecting only some of the inoculated leaves. ND indicates time points not monitored.

ND indicates time points not monitored.

### Quantification of bacterial populations within infected bean plants

**Figure 2:**
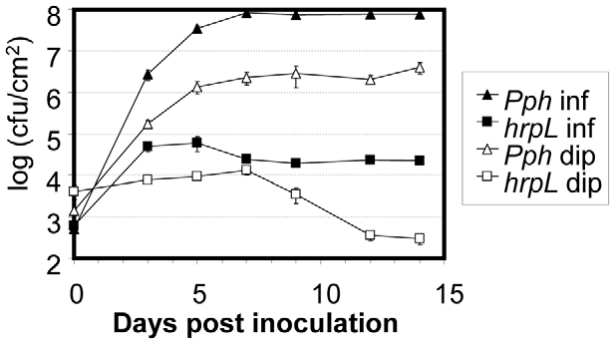
Growth curves of *Pph* 1448a and *Pph* Δ*hrpL*. Bacteria were inoculated either by infiltration with a bacterial suspension containing 5×10^4^ cfu/ml or by dipping into a bacterial suspension containing 5×10^7^ cfu/ml. Error bars represent standard error. Smallest error bars may be covered by the corresponding symbol.

Once inside compatible hosts, *P. syringae* strains replicate rapidly in the apoplastic spaces, leading to the formation of microcolonies and the development of disease [30]. We selected the most relevant time points of this process on the basis of a time course experiment of bean plants (cv. Canadian Wonder) infected with *Pph* 1448a either by infiltration with a blunt syringe, or by dipping the leaves into a bacterial suspension. We included as a non-pathogenic control a Δ*hrpL*-mutant derivative of *Pph* 1448a [31], which lacks HrpL, an essential transcriptional regulator of virulence functions, including the T3SS [32]–[34]. We inoculated the plants by either infiltrating with 5×10^4^ cfu/ml, or dipping into a 5×10^7^ cfu/ml bacterial suspension, in order to achieve an initial population of approximately 1000 cfu per plant one hour after inoculation (t_0_), regardless of the inoculation procedure applied. In the case of *Pph* 1448a, bacterial numbers increased rapidly up to 7 days post-inoculation (dpi), after which the population remained stable up to 14 dpi, with the full onset of symptoms taking place between 10 to 12 dpi (Figure 2 and data not shown). The Δ*hrpL* mutant strain reached its highest populations by 7 dpi, a population 100 to 1000-fold smaller than wild type, and decayed later 5 to 50-fold, depending on the inoculation procedure. As expected, the maximum size of the bacterial population varied depending on the inoculation method (10^8^ cfu/cm^2^ after infiltration and 5×10^6^ cfu/cm^2^ after dip-inoculation), since following dippping, pathogen-associated molecular patterns (PAMPs), like flagellin, are exposed to plant cells before bacteria have reached the apoplast, where full induction of T3SS expression takes place. This allows for an earlier and therefore more effective activation of plant defences using dipping than using infiltration to introduce bacteria directly into the leaf apoplast [35], [36].

Considering these results, we selected for further analysis 4 dpi as a time point in which wild type populations are rapidly growing, while populations of the T3SS-defective mutant still display some growth, and 7 dpi as the time point at which both wild type and mutant populations are stabilized at their respective the maximum cfu. In some cases we also sampled at 14 dpi, to analyse bacterial populations in the context of symptomatic tissue and maximal difference between wild type and mutant populations.

### Several type III effectors from *Pph* 1448a display reduced growth in bean plants

**Figure 3:**
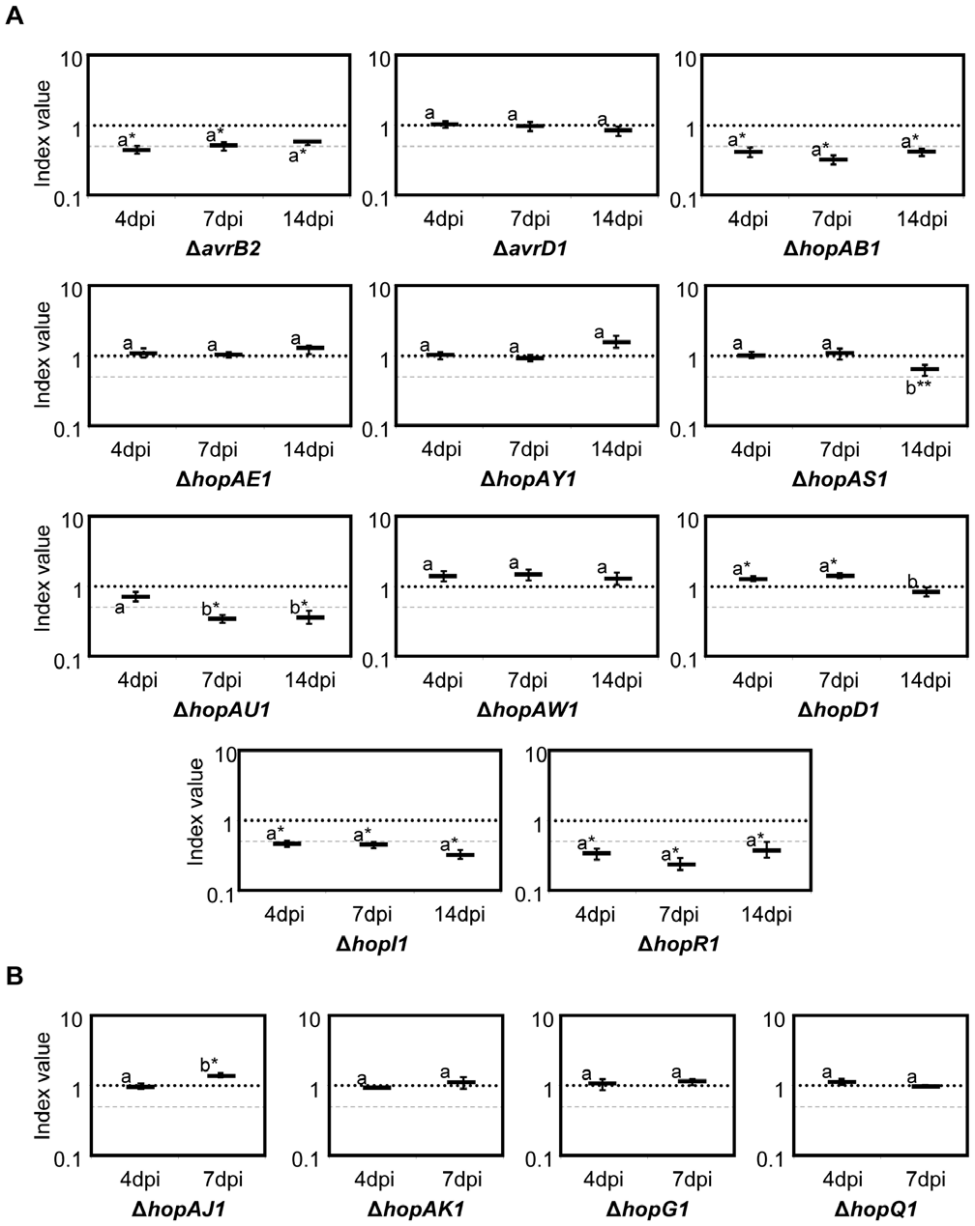
Competitive Index analysis of *Pph* 1448a effector mutants. Mutant strains were co-infiltrated with the wild type strain, and samples were taken at different time points: **A**. 4, 7 and 14 days post-inoculation (dpi), or **B**. 4 and 7 dpi. Competitive indices correspond to the mean of two to four independent experiments with three replicates per experiment. A dashed line corresponding to a CI = 0.5 is included for reference. Error bars represent the standard error. Asterisks indicate results significantly different from one, as established by Student's t-test (P<0.05). Mean values marked with the same letter (a or b) indicate results not significantly different from each other, as established by One Way ANOVA and Holm-Sidak test for multiple comparison or One Way ANOVA on Ranks and Tukey test for multiple comparison when Equal Variance Test failed (*P*<0.05). Two asterisks indicate that attenuation was right on the limit of significance in each independent experiment or when results were pulled from independent experiments.

We applied CI assays to the fifteen effector mutants analysed for development of symptoms (Tables 1 and 2) and determined the CI values at the selected time points after infiltration. Figure 3A shows the results obtained for eleven of these mutants for which CI were determined at 4, 7 and 14 dpi. CI values are shown in Table S1. Five out of them displayed significant virulence attenuation at one or more time points (*ΔavrB2, ΔhopAB1, ΔhopAU1, ΔhopI1, ΔhopR1*; Figure 3A). Interestingly, growth attenuation displayed by *ΔhopAU1* could be detected in fully established populations (7 and 14 dpi), but not at the earliest time point (Figure 3A). The mutant *ΔhopAS1* strain only displayed CI values lower than 1.0 at 14 dpi (Figure 3A), suggesting a contribution to growth at the end of the infection process. However, this attenuation was only statistically significant in two out of three independent experiments and was therefore close to the limit of confidence. No significant attenuation was detected for the other five strains (*ΔavrD1, ΔhopAE1, ΔhopAY1, hopAW1, ΔhopD1*; Figure 3A). It should be mentioned that very small increases in growth (1.3 to 1.5-fold) were detected for *ΔhopAE1* (14 dpi), *ΔhopAY1* (14 dpi), *hopAW1* (4, 7 and 14 dpi), and *ΔhopD1* (4 and 7 dpi) mutant strains, some of which were statistically significant (*ΔhopD1* at both 4 and 7 dpi). However, whether these differences are biologically meaningful is open to question.

The remaining four strains of the collection, *ΔhopG1, ΔhopQ1, ΔhopAJ1* and *ΔhopAK1*, were analysed at 4 and 7 dpi, however no significant attenuation was detected, with only a slight increase in growth (1.5-fold) detectable by 7 dpi for *ΔhopAJ1* (Figure 3B).

**Figure 4:**
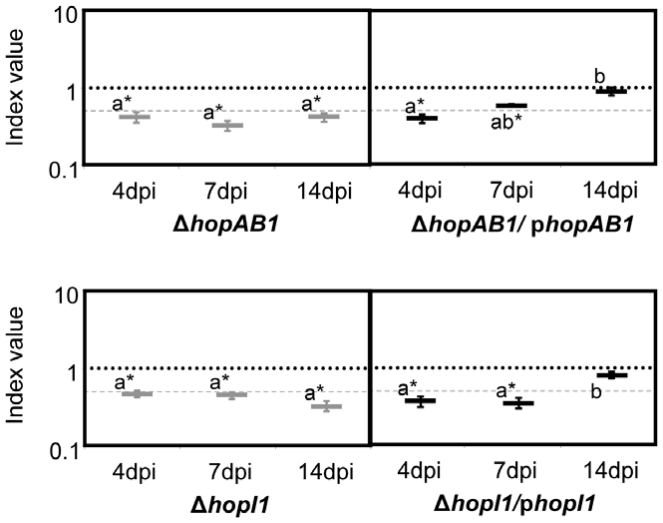
Complementation analysis of Δ*hopAB1* and Δ*hopI1*. The mutant strains carrying a plasmid expressing the corresponding effector from their native promoter were co-inoculated with the wild type strain and samples were taken at different time points (4, 7 and 14 dpi). Competitive indices are the average of three replicates per experiment. The assays were repeated twice with very similar results. The CIs of the relevant mutants are included (grey) for comparison purposes. A dashed line corresponding to a CI = 0.5 is included for reference. Error bars represent the standard error. Asterisks indicate results significantly different from one, as established by Student's t-test (P<0.05). Mean values marked with the same letter (a or b) indicate results not significantly different from each other, as established by One Way ANOVA and Holm-Sidak test for multiple comparison (*P*<0.05).

Additionally, we tested whether the phenotype of *ΔhopAB1* and *ΔhopI1* single mutants, which displayed growth attenuation throughout the course of the infection at a level representative of the majority of the single mutants assayed, could be complemented by plasmid-encoded wild type alleles expressed from the corresponding native promoters. (Figure 3A). By 4 dpi, none of the mutants was complemented since the CI of the mutants and the CI of the mutants carrying the corresponding plasmids were not statistically different (Figure 4). Similar results were observed for *ΔhopI1* at 7 dpi, however, the CI of *ΔhopAB1* carrying the plasmid was significantly higher than that of the mutant strain at that time point, indicating that attenuation of *ΔhopAB1* was being complemented. Nevertheless, complementation was not complete by 7 dpi since the CI of the mutant strain carrying the plasmid was still lower than 1.0 (Figure 4). By 14 dpi, both *ΔhopAB1* and *ΔhopI1* strains were fully complemented by the ectopic expression of the corresponding effector, since the CIs of the mutants carrying the plasmids were not significantly different from 1.0 (Figure 4). All CI values are shown in Table S1. Prior to using the native promoters, we attempted complementation of these two mutant strains by ectopic expression of the effectors from the same vector, but under the control of the medium-to-low expression P*_lacZ_* constitutive promoter: in these conditions no complementation of bacterial growth could be detected at any time point (data not shown).

Considering the potential complexities that validating all attenuated mutant phenotypes through complementation may present on the basis of the results obtained with *ΔhopAB1* and *ΔhopI1*, we analyzed the phenotypes of independently generated knockout mutants for *avrB2*, *hopR1, hopAU1,* and *hopAS1*. CI analysis of all four mutant strains rendered significant attenuations by late stages of the infection (i.e. 14 dpi), but only those from mutants *avrB2* and *hopR1* show significant attenuation by 4 dpi (data not shown), fully confirming previously determined phenotypes.

### Effector overexpression may reduce bacterial growth *in planta*

**Figure 5:**
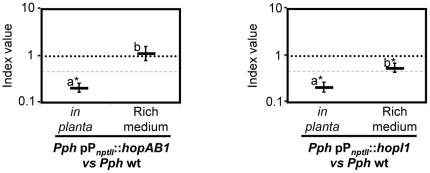
Effect on bacterial growth of overexpressing HopAB1 and HopI1. Wild type strain *Pph* 1448a carrying plasmids expressing either of these effectors from the strong *nptII* promoter were co-inoculated with the wild type strain. Competitive index was analyzed either in bean leaves (at 7 dpi) or in rich medium (LB medium, at 24 hours post-inoculation). Competitive indices are the average of three replicates per experiment. A dashed line corresponding to a CI = 0.5 is included for reference. Error bars represent the standard error. Asterisks indicate results significantly different from one, as established by Student's t-test (P<0.05). Mean values marked with the same letter (a or b) indicate results not significantly different from each other, as established by One Way ANOVA and Holm-Sidak test for multiple comparison or One Way ANOVA on Ranks and Tukey test for multiple comparison when Equal Variance Test failed (*P*<0.05).

The mechanisms by which the *Pph* 1448a effectors analysed contribute to virulence are yet to be determined, but are likely to include suppression of host defences, as described for other strains [37]. Since we found that some of the assayed effectors have a quantitative contribution to *Pph* 1448a bacterial growth *in planta*, we wondered whether increasing their expression could enhance defence suppression and result in an increase in bacterial growth within the plant. We tested HopAB1 and HopI1 since they have homologues in other *P. syringae* pathovars with well-characterised defence suppression capabilities [4], [38]. The *hopAB1* and *hopI1* ORFs were expressed under the control of the strong constitutive P*_nptII_* promoter, from the medium-copy number plasmid pAMEX [29], the backbone vector used for the complementation assays. To our surprise, overexpression of either HopAB1 or HopI1 from *Pph* 1448a caused a clear reduction of growth within the plant compared to the wild type (Figure 5). Growth attenuation due to the overexpression of HopAB1 was specific of growth within the plant, since the CI assay carried out in rich medium was not significantly different to 1.0 (Figure 5). However, overexpression of HopI1 also caused a small reduction in bacterial growth in rich medium, although in this case the reduction was significantly smaller (Figure 5). All CI values are shown in Table S1. These results suggest a need for a tight regulation of effector expression, since effectors otherwise contributing to bacterial growth within the plant may have deleterious effects when expressed beyond their appropriate levels.

### Several effector mutant strains display attenuated growth after dip inoculation

**Figure 6:**
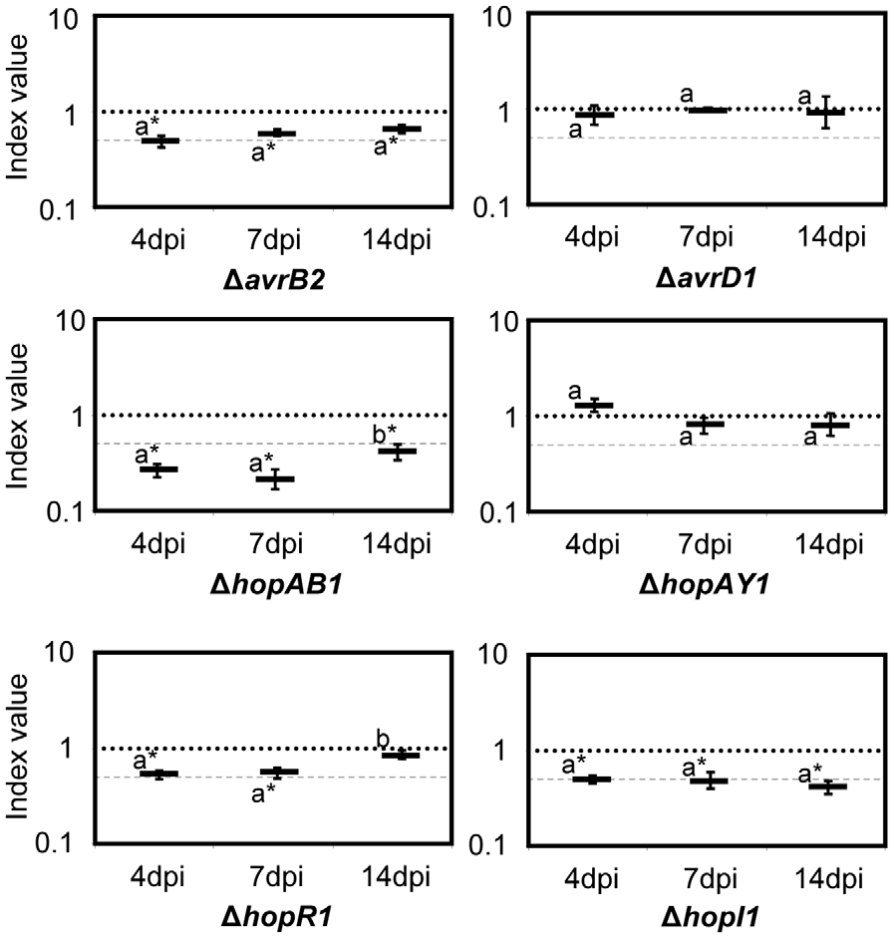
Competitive Index analysis of *Pph* 1448a effector mutants following dip-inoculation. Mutant strains were co-inoculated with the wild type strain, and samples were taken at different time points (4, 7 and 14 days post-inoculation, dpi) as indicated. Competitive indices correspond to the mean of two to three independent experiments with three replicates per experiment. A dashed line corresponding to a CI = 0.5 is included for reference. Error bars represent the standard error. Asterisks indicate results significantly different from one, as established by Student's t-test (P<0.05). Mean values marked with the same letter (a or b) indicate results not significantly different from each other, as established by One Way ANOVA and Holm-Sidak test for multiple comparison (*P*<0.05).

The activation of plant defences through PAMP recognition prior to full induction of the T3SS or bacterial entrance through the stomata determine differences in the pathogen-plant interaction process that may lead to differences in the results obtained from virulence assays following inoculation by infiltration or by dipping [35], [36]. We selected several effector mutants to carry out CI analysis by dipping to determine if this inoculation procedure would lead to different results to those obtained using infiltration. Mutant strains *ΔavrB2, ΔavrD1, ΔhopAB1, ΔhopAY1, ΔhopR1, and ΔhopI1* were co-inoculated with the wild type by dipping and their CIs determined. The CIs obtained were very similar to those obtained using infiltration, except for attenuation of *ΔhopR1* whose attenuation could no longer be established at the end of the infection (14 dpi) (Figure 6). All CI values are shown in Table S1.

### CI-based analysis of functional relationships between effectors

Homologues from three out of the four effectors required for growth throughout the entire course of the infection have defence suppression activities [4], [10], [11], [39]–[41] and, as will be discussed, our virulence assays support similar roles for the corresponding *Pph* 1448a effectors. This raised the question of whether the virulence activities of these four effectors could be functionally related. To established this, we carried out epistasis analysis taking advantage of a CI modification, the cancelled-out index or COI, originally set up for such purpose in animal pathogens [18], [42]–[45], and recently applied to the analysis of structural and regulatory components of the T3SS in *Pph* 1448a [19], [20], and to that of plant defence responses against *Pto* DC3000 expressing heterologous effectors [21], [22]. The question to be answered by COI-based epistasis analysis was whether the growth attenuation phenotype caused by Δ*hopAB1* could be detected in the absence of any of the other two effectors. For that purpose, double mutant strains lacking HopAB1 and each of the other effectors (HopR1, and HopI1) were generated by introducing the knockout alleles for *hopR1* and *hopI1* in the Δ*hopAB1* single mutant strain. Double mutant strains were co-inoculated with each of the corresponding single mutant strains. The index generated from calculating the double to single mutant output ratio, divided by the corresponding input ratio, the cancelled out index, allows direct determination of the phenotype that mutation of *hopAB1* has in the absence of each of the other effectors (Figure S1). Thus, each of the effectors analysed is missing both in the single and the double co-inoculated mutants, and its effect in growth is thus cancelled out in the resulting index. If the phenotype of Δ*hopAB1* cannot be established in the absence of the other effectors the resulting COI would be equal to one (Figure S1). Reversely, if HopAB1 contribution to virulence can be detected in the absence of the effector tested, Δ*hopAB1* would have the same phenotype regardless of the genotype of the effector gene being tested, and the resulting COI would therefore be equal to its CI (Figure S1). Controls to confirm that both types of results (COI not significantly different from 1.0 and COI not significantly different from the CI) could be obtained when analysing effector mutants with such subtle attenuated phenotypes were carried out (Figure S2). COI values are shown in Table S1.

**Figure 7:**
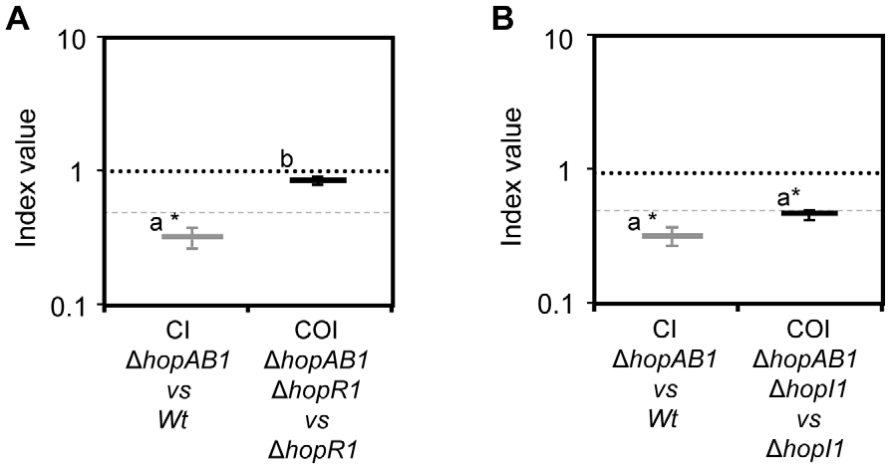
Cancelled-out index (COI) epistasis analysis of *Pph* 1448a effectors. Epistasis analysis of HopR1 (A) and HopI1 (B) on the contribution to virulence of HopAB1. Double mutants strains were co-infiltrated with each single mutant strain and the corresponding COI was determined at 7 dpi. All relevant CIs are included (grey) for comparison purposes. Each COI corresponds to the mean of at least three independent experiments with three replicates per experiment. A dashed line corresponding to an index value = 0.5 is included for reference. Error bars represent the standard error. Asterisks indicate results significantly different from one, as established by Student's t-test (*P*<0.05). Mean values marked with the same letter (a or b) indicate results not significantly different from each other, as established by One Way ANOVA and Holm-Sidak test for multiple comparisons (*P*<0.05).

COI-based epistasis analysis showed that whereas Δ*hopAB1* phenotype could not be detected when HopR1 was also missing (COI of Δ*hopAB1* when Δ*hopR1* is cancelled out is not significantly different from 1.0, Figure 7A), it was fully detected when HopI1 was missing (COI of Δ*hopAB1* when Δ*hopI1* is cancelled out is not significantly different from the CI of Δ*hopAB1*, Figure 7B). Both results were confirmed by analysing independently generated double mutant strains (data not shown).

When the reciprocal analyses were carried out, we found that whereas HopR1 contribution to virulence was independent on the function of HopAB1, HopI1 contribution was largely independent from HopAB1 (COI showed a 1.5-fold attenuation *versus* >3-fold shown by CI) (Table 3).

**Table 3:** Epistatic analysis of HopAB1 function on the contribution to virulence of HopR1 and HopI1.

Time point	Knockout effector tested	COI (when Δ*hopAB1 is* cancelled out)	CI	COI different from CI?	COI different from 1.0?
7 dpi	Δ*hopR1*	0.32+/−0.003	0.24+/−0.05	No	Yes
7 dpi	Δ*hopI1*	0.59+/−0.11	0.46+/−0.05	No	Yes

## Discussion

The individual contribution of T3SS effectors to virulence has been traditionally difficult to establish in *P. syringae*, a fact usually explained by the redundant activities reported for several functionally characterized effectors [3]–[7]. However, our previous results showed that even effectors proven to be functionally redundant could display an individual contribution to virulence if sensitive assays such as CI assays were used [15]. Indeed, the use of CI assays to analyse effector mutants in *Pph* 1448a has allowed us to establish a role in virulence for six out of the fifteen effectors analysed. This represents a higher percentage of effectors with established individual contributions to virulence within the *Pph* 1448a effector inventory than any previously studied *P. syringae* strain [10], [14]. Whether this is due to differences in the composition of the effector inventories analysed or to the different methodology used in each study is yet to be determined, although our previous results with AvrPto1*_Pto_*_DC3000_ [15] supports the latter. Although the contribution of individual effectors to virulence is quite small in laboratory conditions, their relative importance for the development of disease is likely to be larger in the field, where conditions are not as favourable for establishing infection as they are following forced inoculation in the laboratory.

The CI assays carried out in bean allows us to classify *Pph* 1448a effectors into three groups: group I effectors, including those individually required for wild type-like growth throughout the course of the infection (AvrB2, HopAB1, HopI1, and HopR1); group II effectors, including those individually necessary for growth at later stages of the infection (HopAS1 and HopAU1); and group III including effectors and/or helper proteins not individually necessary for growth within bean leaves (AvrD1, HopAE1, HopAY1, HopAW1, HopD1, HopAJ1, HopAK1, HopG1 and HopQ1).

The absence of a growth attenuation phenotype for mutants of Group III proteins could be caused by their having completely redundant activities, such as it is the case for AvrB4-1 and AvrB4-2, or HopW1-1 and HopW1-2, for which the use of double mutants is necessary to detect attenuation of growth by CI assays [17]. It is also possible that the contribution to virulence of these proteins is more evident in other host and/or field conditions. To this regard, direct infiltration into the leaf apoplast could potentially hinder detection of virulence contribution by bypassing the initial steps of the natural infection. However the CI analysis using dip-inoculation of at least two of these effectors (AvrD1 and HopAY1) did not support this notion. Finally, some of these effectors could trigger defence responses that could mask their virulence activities. Our results show that loss of five of the nine Group III proteins result in enhanced growth, which could support this notion, however this increase was only statistically significant for two of the cases and even then the level of enhancement was so low (1.3 to 1.5-fold) as to be difficult to establish their biological significance.

Contribution to virulence for group II effectors (HopAU1 and HopAS1) is only apparent at late and/or very late stages of the infection process, preventing the mutant populations from reaching wild type levels. These effectors could be involved in modifying the apoplast into a replication-permissive niche, where a full colony can develop, rather than in early suppression of plant defences.

It is noteworthy that whereas effectors belonging to group I are present in 60% to almost 100% of the phytopathogenic bacterial strains sequenced to date, those belonging to groups II and III are only present in 20% to less than 50% of the sequenced strains, with the exception of HopAE1, HopAJ1, and HopAK1 [46], although whether HopAJ1 and HopAK1 act as effectors or as helpers of translocation is still unclear [27]–[29]. Furthermore, very little information is available on the virulence or avirulence activities of Group II and III effectors, whereas homologues for all four Group I effectors have been characterized to some level, supporting the relevance of the virulence activities carried out by effectors from Group I. To this regard, the *Pto* DC3000 homologue of HopAB1 (also known as AvrPtoB*_Pto_*_DC3000_), the homologue of HopI1 from *P. syringae* pv. *maculicola* (*Pma* ES4326), and at least one member of each of the families that form the AvrE/DspA/E/HopM1/HopR superfamily, have been shown to promote bacterial growth [4], [16], [47]–[51]. The AvrB family has been reported to have a small but significant contribution to bacterial growth in compatible interactions and a contribution to leaf chlorosis in Arabidopsis in the absence of RPM1 recognition, although whether this chlorosis is the result of virulence or avirulence activities is still unclear [52]–[54]. Members of the HopR1, HopI1 and HopAB1 effector families have all been shown to have some defence suppression activity. HopR1*_Pto_*_DC3000_, which displays 85% amino acid identity with its *Pph* 1448a homologue, has been shown to function redundantly with other effectors in promoting bacterial growth by suppressing PAMP-triggered immunity [10], [40]. HopI1*_Pma_*_ES4326_ hijacks the plant chaperone machinery, causing chloroplast thylakoid structure remodelling and suppressing SA accumulation [4], [39]. HopI1*_Pph_*_1448a_ shares 68% amino acid identity with HopI1*_Pma_*_ES4326_ and although shorter by virtue of some amino acids missing from its middle section, still maintains a putative J domain in its C-terminal region (Figure S3), a domain necessary for HopI1*_Pma_*_ES4326_ contribution to virulence [4]. Finally, HopAB1*_Pph_*_1449b_ (formerly VirPphA) was the first effector for which a virulence activity was demonstrated, postulated to be suppression of HopF1 (formerly AvrPphF)-triggered immunity [41]. HopAB1 orthologs from different *P. syringae* pathovars have been since described to suppress and/or trigger defences in different plant species [38], [47]. HopAB2*_Pto_*_DC3000_, also known as AvrPtoB*_Pto_*_DC3000_, harbours a C-terminal E3 ubiquitin ligase domain [55], which specifically ubiquitinates the Fen kinase in tomato plants, promoting its proteasome-dependent degradation [56]. This activity prevents plant defence activation through the recognition by the Fen kinase of the HopAB2*_Pto_*_DC3000_ N-terminal domain [56]. HopAB1*_Pph_*_1448a_ amino acid sequence is 100% identical to that from *Pph* 1449b strain (data not shown), and both have a predicted E3 ligase C-terminal domain (Figure S3). Virulence activities similar to those described by their homologues would explain HopR1, HopI1 and HopAB1 contribution to virulence, and are in keeping with their protein similarities. In support of this role for HopAB1, HopAB2*_Pto_*_DC3000_ has been described to mimic HopAB1*_Pph_*_1449b_ promotion of virulence when expressed from a *Pph* 1449b lacking HopAB1*_Pph_*_1449b_ infecting bean resistant cultivars, and that deletions in its C-terminal domain abolish this activity [47].

Overexpression of both HopI1_Pph1448a_ and HopAB1_Pph1448a_ cause a reduction of bacterial growth *in planta*. Expression of these effectors could require a tight regulation to avoid unspecific alterations of plant processes (*i.e.* chloroplast activity or unspecific ubiquitin-directed, proteasome-mediated protein degradation), or could alter the secretion hierarchy. However, in the case of HopI1, the slight reduction of growth that also determines in rich medium suggests an additional toxic effect for the bacteria. A requirement for a tight regulation of expression for some effectors is supported by our complementation results (Figure 4; [17]). Expression of HopAB1 and HopI1 only complements the mutant phenotypes when expression is driven from their native promoters, and not from P*_lacZ_*, and even in this case complementation can only be detected at late stages of the infection, suggesting that either *in trans* expression, or differences in gene dosage prevent full complementation. We also showed previously that growth attenuation of a Δ*avrB4*-*1* Δ*avrB4*-*2* double mutant could not be complemented when either AvrB4-1 or AvrB4-2 was expressed from P*_nptII_*, but was complemented by either AvrB4-1 expression from its native promoter, or P*l_acZ_*-driven AvrB4-2 expression.

Since three out of the four group I effectors have homologues with demonstrated defence suppression capabilities, and both their mutant phenotypes within the plant (Figure 3), and the protein similarities to their homologues supports similar activities for the *Pph* 1448a effectors (Figure S3), we carried out epistasis analysis to establish their mutual functional relationships. Our results show that HopAB1 contribution to virulence is independent from the function of HopI1 and that of HopI1 is also independent from HopAB1 function. Thus, we believe these effectors do contribute to *Pph* 1448a virulence independently. Information available for the virulence activities of their homologues in other pathovars does support this notion. Reversely, HopAB1 contribution to virulence is fully dependent on the function of HopR1, whereas that of HopR1 is independent from HopAB1 function. These results fit a model in which both of these effectors would contribute to virulence by suppressing at different levels the same or related defence pathways, in such a manner that for HopAB1-mediated suppression to be effective, HopR1 suppression must take place.

Further analysis would be necessary to establish the molecular basis for the functional interactions detected between these effectors. Our results highlight the potential diversity of the functional relationships displayed by effectors encoded by a given pathogen. It also highlights the need to study effector function within the context of the infection, where the virulence of a given strain is not a simple summation of the functions performed by each effector when individually assayed in heterologous systems, but the complex result of their mutual functional interactions. We also believe that this study also proves the potential that highly sensitive virulence assays such as mixed infection-based assays (i.e. CI and COI) have in the challenge of understanding how the different effector activities of a given pathogen participate and relate to determines virulence, particularly when coupled with classical genetic analysis.

## Materials and Methods

### Bacterial strains and growth conditions

Bacterial strains used in this work are listed in Table 1. *Pph* 1448a and derivative strains were grown at 28°C in Luria-Bertani (LB) medium. Antibiotics were used at the following concentration: kanamycin (15 µg/ml), rifampicin (15 µg/ml), and cycloheximide (2 µg/ml).

### Plasmids and cloning procedures

To obtain p*hopAB1* and p*hopI1*, the ORFs of *hopAB1* and *hopI1,* including their putative ribosome-binding site and their native promoter, were PCR-amplified and cloned into pBBR1MCS4 [29], [57], using *Pph* 1448a genomic DNA as a template, and the corresponding primers (full_*hopAB1*-R 5′-GTCGAATTCCCATACGGTAATGTTGACCC-3′, full_*hopAB1*-F 5′-GTCAAGCTTGTCAAACAGCAACTATTGGG-3′, *hopI1*-R 5′-GTCGAATTCTTCTGACAGTCTCCTCACGC-3′ and full_*hopI1*-F 5′-GTCAAGCTTGCACACACCTGACTGATGC-3′). To generate pP*_nptII_*::*hopAB1* and pP*_nptII_*::*hopI1*, the ORFs of *hopAB1* and *hopI1*, excluding their native promoter but not their ribosome-binding sites, were PCR-amplified and cloned into pAMEX [29] under the control of the P*_nptII_* promoter, using *Pph* 1448a genomic DNA as a template, and the corresponding primers (*hopAB1*-R 5′-GTCGAATTCCGATGCTCTCTTGAAAAACGG-3′, *hopAB1*-F 5′-GTCAAGCTTTTCGCAACCATGAGATCAGG-3′, *hopI1*-R 5′- GTCGAATTCTTCTGACAGTCTCCTCACGC-3′ and *hopI1*-F 5′-GTCAAGCTTACTAGATCCCGTTGCTTGCC-3′).s

### Generation of double mutant strains

Double mutant strains (Table 1) were generated as follows. Plasmid pFLP2, expressing the flipase enzyme, was transformed into the corresponding single mutant strain, to promote removal of the *nptII* gene by flipase-mediated site-specific recombination. Transformants were tested in LB plates with kanamycin to identify clones in which the kanamycin gene had been removed. Kanamycin-sensitive isolates were then grown in LB plates supplemented with 5% sucrose to select those that had lost the pFLP2 vector. A second allelic exchange vector was then transformed into the resulting kanamycin-sensitive single knockout strain, and transformants were selected and analyzed as described [17].

### Competitive index, cancelled-out index, and standard growth assays

CI assays in bean plants (*Phaseolus vulgaris* cv. Canadian wonder) were carried out as previously described [15]. For inoculations by infiltration, 8-days old bean plants, grown at 22°C to 28°C with a photoperiod of 16/8 h light/dark cycle, were inoculated with 200 µl of a 5×10^4^ cfu/ml mixed bacterial suspension in 10 mM MgCl_2_, containing equal cfu of wild type and mutant or gene-expressing strain, using a 1 ml syringe without needle. For inoculations by dipping, leaves were dipped for 30 seconds in a 5×10^7^ cfu/ml mixed bacterial suspension in 10 mM MgCl_2_ and 0.02% Silwett L-77 (Crompton Europe Ltd, Evesham, UK). Serial dilutions of the inoculum were plated onto LB agar and LB agar with the appropriate antibiotic to confirm bacteria cfu relative proportion between the strains, which should be close to one. At different days post-inoculation (dpi), bacteria were recovered from the inoculated leaves. Bacterial recovery was carried out by taking five 10 mm-diameter discs with a cork-borer, which were homogenized by mechanical disruption into 1 ml of 10 mM MgCl_2_. Bacterial enumeration was performed by serial dilution and plating of the samples onto agar plates with cycloheximide and the appropriate antibiotic to differentiate the strains within the mixed infection. For standard replication assays, the same inoculation procedure was carried out using an individual instead of a mixed inoculum.

Five hundred µl of a mixed inoculum at 5×10^4^ cfu/ml, containing equal amounts of wild type and mutant bacteria was inoculated into 4.5 ml of LB medium and grown for 24 h at 28°C with aeration to determine ^LB^CIs. Serial dilutions were then plated onto LB-agar and LB with the corresponding antibiotic to calculate the relative proportion between the strains.

The CI is defined as the mutant-to-wild type ratio within the output sample divided by the mutant-to-wild type ratio within the input (inoculum) [58], [59]. Cancelled out index (COI) was determined using the same methodology but co-inoculating a single with a double mutant strain. COI is defined as the double mutant-to-single mutant ratio within the output sample divided by the double mutant-to-single mutant ratio within the input (inoculum) [18], [44].

Competitive and cancelled out indices presented are the mean of at least three independent experiments with three replicates per experiment. Errors bars represent standard error. Each CI or COI was analyzed using a homoscedastic and 2-tailed Student's t-test and the null hypothesis: mean index is not significantly different from 1.0 (P value <0.05). CI or COI comparisons were tested for statistical significance by One Way ANOVA and Holm-Sidak test for multiple comparison or One Way ANOVA on Ranks and Tukey test for multiple comparison when Equal Variance Test failed (*P*<0.05).

## Supporting Information

Figure S1 **Cancelled-out index (COI) analysis of** ***Pph*** **1448a effectors HopAB1 and HopQ1.** The double mutants strains were co-infiltrated with each single mutant strain and the corresponding COI was determined at either at 7 dpi. Each relevant CIs is included in the figure (grey) for comparison purposes. Each COI corresponds to the mean of at least three independent experiments with three replicates per experiment. A dashed line corresponding to an index value = 0.5 is included for reference. Error bars represent the standard error. Asterisks indicate results significantly different from one, as established by Student's t-test (*P*<0.05). Mean values marked with the same letter (a or b) indicate results not significantly different from each other, as established by One Way ANOVA and Holm-Sidak test for multiple comparisons (*P*<0.05).
(TIF)

Figure S2 **Theoretical representation of COI analysis of the interaction between two hypothetical genes,** ***a*** **and** ***b*** **.** **A.** CI is defined as the mutant-to-wt output ratio divided by the mutant-to-wt input ratio. COI is defined as the double mutant-to-single mutant output ratio divided by the double mutant-to-single mutant input ratio. **B.** Determination and analysis of COI. A mix inoculum containing an equal bacterial number of double and single mutant stains is infiltrate into plant leaves. Bacteria are recovered from plant leaves 4, 7 or 14 days post inoculation (dpi), and plated into LB and LB supplemented with antibiotics, to differentiate double and single mutants. I and II represent two possible outcomes for the analysis. CI^a^ is a CI of a strain carrying a mutation in gene *a* co-inoculated with the wt strain. COI^a^ is a COI of a strain carrying a mutation in gene *b* co-inoculated with the double mutant strain.
(TIF)

Figure S3 **Sequence analysis of effectors HopAB1 and HopI1.** **A.** Comparison of HopAB1_Pph1448a_ and AvrPtoB_PtoDC3000_ amino acid sequences. Identical amino acids are highlighted in blue. Sequences display 55% overall identity, while the C-terminal region predicted to comprise the E3 ligase domain (Pfam ID: PF09046, underlined), displays 77% identity. Position of conserved prolines (AvrPtoB_Pro533_, HopAB1_Pro519_) and large hydrophobic residues (AvrPtoB_Phe479, Phe525_, HopAB1_Phe465, Tyr511_) described as essential for E3 ligases [51] are marked by red arrows. **B.** Comparison of *Pph* 1448a and *Pma* ES4326 HopI1 amino acid sequences. Identical amino acids are highlighted in blue. Predicted J domain (Pfam ID: PF00226; Prosite ID: PS50076) is underlined. Sequences display 68% overall identity.
(TIF)

Table S1 Numerical values corresponding for the CIs and COIs displayed in figure 3, 4, 5, 6, 7 and S1.
(DOC)
