# Supplementary figures and images for: Genetic Analysis of the Individual Contribution to Virulence of the Type III Effector Inventory of Pseudomonas syringae pv. phaseolicola
Source: PLoS One. 2012 Apr 27;7(4):e35871. doi: 10.1371/journal.pone.0035871 (PMC3338808; doi:10.1371/journal.pone.0035871)

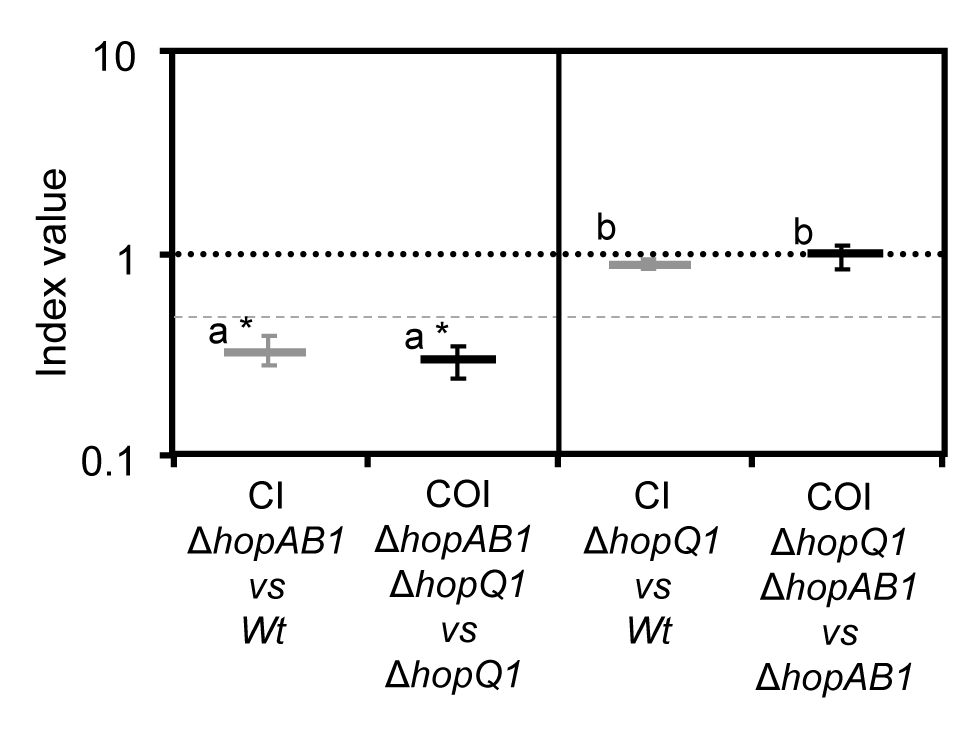

Supplement: Figure S1 — Cancelled-out index (COI) analysis of Pph 1448a effectors HopAB1 and HopQ1. The double mutants strains were co-infiltrated with each single mutant strain and the corresponding COI was determined at either at 7 dpi. Each relevant CIs is included in the figure (grey) for comparison purposes. Each COI corresponds to the mean of at least three independent experiments with three replicates per experiment. A dashed line corresponding to an index value = 0.5 is included for reference. Error bars represent the standard error. Asterisks indicate results significantly different from one, as established by Student's t-test (P<0.05). Mean values marked with the same letter (a or b) indicate results not significantly different from each other, as established by One Way ANOVA and Holm-Sidak test for multiple comparisons (P<0.05). (TIF) [file pone.0035871.s001.tif]

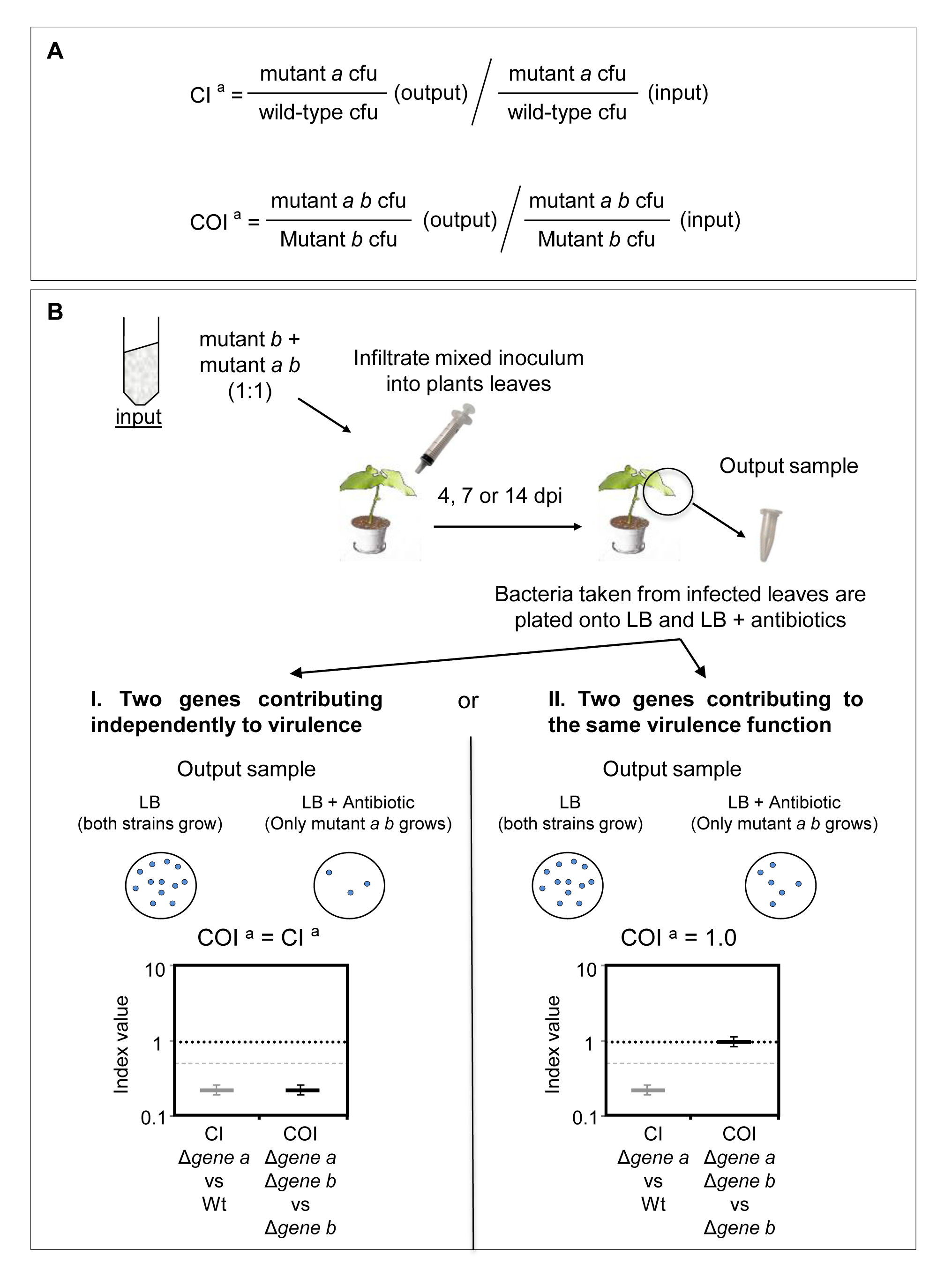

Supplement: Figure S2 — Theoretical representation of COI analysis of the interaction between two hypothetical genes, a and b . A. CI is defined as the mutant-to-wt output ratio divided by the mutant-to-wt input ratio. COI is defined as the double mutant-to-single mutant output ratio divided by the double mutant-to-single mutant input ratio. B. Determination and analysis of COI. A mix inoculum containing an equal bacterial number of double and single mutant stains is infiltrate into plant leaves. Bacteria are recovered from plant leaves 4, 7 or 14 days post inoculation (dpi), and plated into LB and LB supplemented with antibiotics, to differentiate double and single mutants. I and II represent two possible outcomes for the analysis. CIa is a CI of a strain carrying a mutation in gene a co-inoculated with the wt strain. COIa is a COI of a strain carrying a mutation in gene b co-inoculated with the double mutant strain. (TIF) [file pone.0035871.s002.tif]

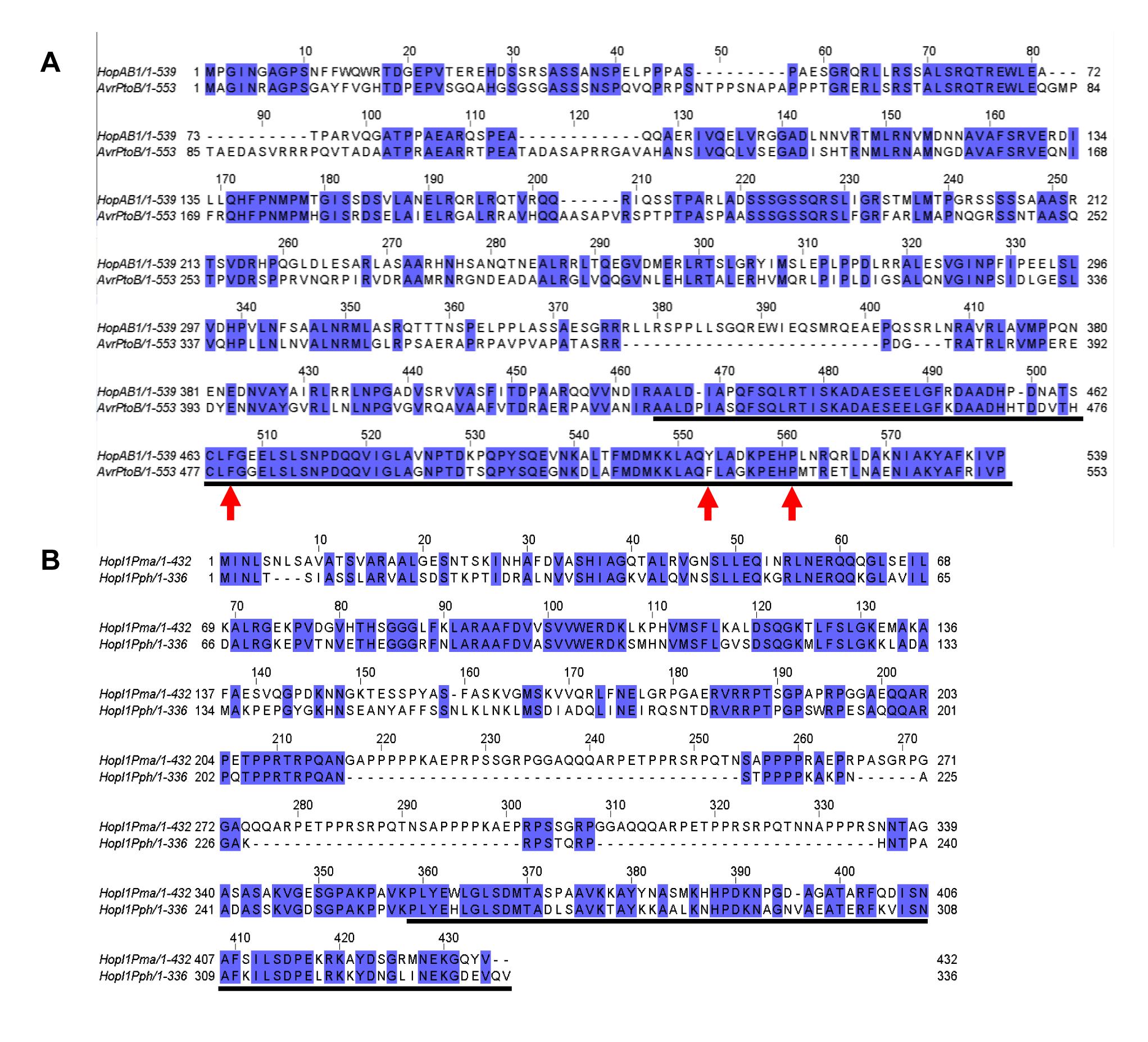

Supplement: Figure S3 — Sequence analysis of effectors HopAB1 and HopI1. A. Comparison of HopAB1Pph1448a and AvrPtoBPtoDC3000 amino acid sequences. Identical amino acids are highlighted in blue. Sequences display 55% overall identity, while the C-terminal region predicted to comprise the E3 ligase domain (Pfam ID: PF09046, underlined), displays 77% identity. Position of conserved prolines (AvrPtoBPro533, HopAB1Pro519) and large hydrophobic residues (AvrPtoBPhe479, Phe525, HopAB1Phe465, Tyr511) described as essential for E3 ligases [51] are marked by red arrows. B. Comparison of Pph 1448a and Pma ES4326 HopI1 amino acid sequences. Identical amino acids are highlighted in blue. Predicted J domain (Pfam ID: PF00226; Prosite ID: PS50076) is underlined. Sequences display 68% overall identity. (TIF) [file pone.0035871.s003.tif]
