# Supplementary material for: Genetic Analysis of the Individual Contribution to Virulence of the Type III Effector Inventory of Pseudomonas syringae pv. phaseolicola
Source: PLoS One. 2012 Apr 27;7(4):e35871. doi: 10.1371/journal.pone.0035871 (PMC3338808; doi:10.1371/journal.pone.0035871)
Supplement: Table S1 — Numerical values corresponding for the CIs and COIs displayed in figure 3, 4, 5, 6, 7 and S1. (DOC) [file pone.0035871.s004.doc]

**Supplementary table 1**.

| **Time point** | **Knockout effector tested** | **CI after infiltration** | **CI different from 1?** |
| --- | --- | --- | --- |
| 4 dpi | Δ*hopAB1* | 0.43+/-0.07 | Yes |
| 7 dpi | Δ*hopAB1* | 0.32+/-0.05 | Yes |
| 14 dpi | Δ*hopAB1* | 0.44+/-0.06 | Yes |
| 4 dpi | Δ*hopR1* | 0.35+/-0.07 | Yes |
| 7 dpi | Δ*hopR1* | 0.24+/-0.05 | Yes |
| 14 dpi | Δ*hopR1* | 0.41+/-0.10 | Yes |
| 4 dpi | Δ*hopI1* | 0.49+/-0.05 | Yes |
| 7 dpi | Δ*hopI1* | 0.46+/-0.05 | Yes |
| 14 dpi | Δ*hopI1* | 0.33+/-0.05 | Yes |
| 4 dpi | Δ*hopAS1* | 1.09+/-0.10 | No |
| 7 dpi | Δ*hopAS1* | 1.16+/-0.23 | No |
| 14 dpi | Δ*hopAS1* | 0.67+/-0.12 | Yes/No^a^ |
| 4 dpi | Δ*hopAU1* | 0.77+/-0.12 | No |
| 7 dpi | Δ*hopAU1* | 0.37+/-0.04 | Yes |
| 14 dpi | Δ*hopAU1* | 0.37+/-0.08 | Yes |
| 4 dpi | Δ*avrB2* | 0.46+/-0.04 | Yes |
| 7 dpi | Δ*avrB2* | 0.52+/-0.07 | Yes |
| 14 dpi | Δ*avrB2* | 0.59+/-0.02 | Yes |
| 4 dpi | Δ*avrD1* | 1.06+/-0.11 | No |
| 7 dpi | Δ*avrD1* | 1.03+/-0.15 | No |
| 14 dpi | Δ*avrD1* | 0.87+/-0.12 | No |
| 4 dpi | Δ*hopAE1* | 1.15+/-0.18 | No |
| 7 dpi | Δ*hopAE1* | 1.08+/-0.11 | No |
| 14 dpi | Δ*hopAE1* | 1.30+/-0.17 | No |
| 4 dpi | Δ*hopAY1* | 1.05+/-0.15 | No |
| 7 dpi | Δ*hopAY1* | 0.98+/-0.12 | No |
| 14 dpi | Δ*hopAY1* | 1.60+/-0.33 | No |
| 4 dpi | Δ*hopAW1* | 1.49+/-0.21 | No |
| 7 dpi | Δ*hopAW1* | 1.52+/-0.28 | No |
| 14 dpi | Δ*hopAW1* | 1.35+/-0.30 | No |
| 4 dpi | Δ*hopD1* | 1.31+/-0.13 | Yes |
| 7 dpi | Δ*hopD1* | 1.46+/-0.13 | Yes |
| 14 dpi | Δ*hopD1* | 0.87+/-0.12 | No |
| 4 dpi | Δ*hopQ1* | 1.20+/-0.12 | No |
| 7 dpi | Δ*hopQ1* | 0.96+/-0.04 | No |
| 4 dpi | Δ*hopAJ1* | 1.01+/-0.09 | No |
| 7 dpi | Δ*hopAJ1* | 1.51+/-0.14 | Yes |
| 4 dpi | Δ*hopAK1* | 0.98+/-0.03 | No |
| 7 dpi | Δ*hopAK1* | 1.20+/-0.22 | No |
| 4 dpi | Δ*hopG1* | 1.14+/-0.21 | No |
| 7 dpi | Δ*hopG1* | 1.21+/-0.13 | No |
| ^a^Independent experiments with similar CI values were significantly different to 1.0 or not depending on the standard error of each | | | |
| **Time point** | **Effector overexpressed** | **CI after infiltration** | **CI different from 1?** |
| 7 dpi | HopAB1 | 0.19+/-0.05 | Yes |
| 7 dpi | HopI1 | 0.22+/-0.05 | Yes |
| **Time point** | **Effector overexpressed** | **CI *in vitro*** | **CI different from 1?** |
| 24 h | HopAB1 | 1.20+/-0.39 | No |
| 24 h | HopI1 | 0.59+/-0.13 | Yes |
| **Time point** | **Knockout effector tested** | **CI after dip inoculation** | **CI different from 1?** |
| 4 dpi | Δ*hopAB1* | 0.27+/-0.04 | Yes |
| 7 dpi | Δ*hopAB1* | 0.22+/-0.05 | Yes |
| 14 dpi | Δ*hopAB1* | 0.44+/-0.08 | Yes |
| 4 dpi | Δ*hopR1* | 0.57+/-0.05 | Yes |
| 7 dpi | Δ*hopR1* | 0.59+/-0.08 | Yes |
| 14 dpi | Δ*hopR1* | 0.90+/-0.09 | No |
| 4 dpi | Δ*hopI1* | 0.54+/-0.06 | Yes |
| 7 dpi | Δ*hopI1* | 0.52+/-0.12 | Yes |
| 14 dpi | Δ*hopI1* | 0.44+/-0.06 | Yes |
| 4 dpi | Δ*avrB2* | 0.53+/-0.07 | Yes |
| 7 dpi | Δ*avrB2* | 0.63+/-0.04 | Yes |
| 14 dpi | Δ*avrB2* | 0.69+/-0.08 | Yes |
| 4 dpi | Δ*avrD1* | 0.87+/-0.20 | No |
| 7 dpi | Δ*avrD1* | 1.00+/-0.04 | No |
| 14 dpi | Δ*avrD1* | 0.98+/-0.36 | No |
| 4 dpi | Δ*hopAY1* | 1.39+/-0.19 | No |
| 7 dpi | Δ*hopAY1* | 0.85+/-0.15 | No |
| 14 dpi | Δ*hopAY1* | 0.89+/-0.23 | No |

| **Time point** | **Effector complementation**  (effector expressed from their native promoter) | **CI after infiltration** | **CI different from CI mutant?** | **CI different from 1?** |
| --- | --- | --- | --- | --- |
| 4 dpi | Δ*hopAB1*/HopAB1 | 0.43+/-0.06 | No | Yes |
| 7 dpi | Δ*hopAB1*/HopAB1 | 0.76+/-0.02 | Yes | Yes |
| 14 dpi | Δ*hopAB1*/HopAB1 | 0.94+/-0.12 | Yes | No |
| 4 dpi | Δ*hopI1*/HopI1 | 0.37+/-0.06 | No | Yes |
| 7 dpi | Δ*hopI1*/HopI1 | 0.36+/-0.05 | No | Yes |
| 14 dpi | Δ*hopI1*/HopI1 | 0.84+/-0.08 | Yes | No |

| **Time**  **point** | **Knockout effector tested** | **COI** | **CI** | **COI different from CI?** | **COI different from 1.0?** |
| --- | --- | --- | --- | --- | --- |
| 7 dpi | ΔhopAB1  (when Δ*hopR1 is* cancelled out) | 0.88+/-0.06 | 0.32+/-0.05 | Yes | No |
| 7 dpi | ΔhopAB1  (when Δ*hopI1 is* cancelled out) | 0.48+/-0.04 | 0.32+/-0.05 | No | Yes |
| 7 dpi | ΔhopQ1  (when Δ*hopAB1 is* cancelled out) | 0.97+/-0.15 | 0.96+/-0.04 | No | No |
| 7 dpi | ΔhopAB1  (when Δ*hopQ1 is* cancelled out) | 0.31+/-0.06 | 0.32+/-0.05 | No | Yes |
